# Inbreeding Effects on the Performance and Genomic Prediction for Polysomic Tetraploid Potato Offspring Grown at High Nordic Latitudes

**DOI:** 10.3390/genes14061302

**Published:** 2023-06-20

**Authors:** Rodomiro Ortiz, Fredrik Reslow, Ramesh Vetukuri, M. Rosario García-Gil, Paulino Pérez-Rodríguez, José Crossa

**Affiliations:** 1 Department of Plant Breeding, Swedish University of Agricultural Sciences (SLU), SE 23436 Lomma, Sweden; 2 Umeå Plant Science Center, SLU Department of Forest Genetics and Plant Physiology, Swedish University of Agricultural Sciences (SLU), SE 90183 Umeå, Sweden; m.rosario.garcia@slu.se; 3 Colegio de Postgraduados (COLPOS), Montecillos 56230, Edo. de México, Mexico; perpdgo@gmail.com (P.P.-R.); j.crossa@cgiar.org (J.C.); 4 International Maize and Wheat Improvement Center (CIMMYT), El Batán, Texcoco 56237, Edo. de México, Mexico

**Keywords:** *Solanum tuberosum* L., accuracy, Fennoscandia, GEBV, genetic gains, germplasm enhancement, hybrid, inbred, QTL, Scandinavia

## Abstract

Inbreeding depression (ID) is caused by increased homozygosity in the offspring after selfing. Although the self-compatible, highly heterozygous, tetrasomic polyploid potato (*Solanum tuberosum* L.) suffers from ID, some argue that the potential genetic gains from using inbred lines in a sexual propagation system of potato are too large to be ignored. The aim of this research was to assess the effects of inbreeding on potato offspring performance under a high latitude and the accuracy of the genomic prediction of breeding values (GEBVs) for further use in selection. Four inbred (S_1_) and two hybrid (F_1_) offspring and their parents (S_0_) were used in the experiment, with a field layout of an augmented design with the four S_0_ replicated in nine incomplete blocks comprising 100, four-plant plots at Umeå (63°49′30″ N 20°15′50″ E), Sweden. S_0_ was significantly (*p* < 0.01) better than both S_1_ and F_1_ offspring for tuber weight (total and according to five grading sizes), tuber shape and size uniformity, tuber eye depth and reducing sugars in the tuber flesh, while F_1_ was significantly (*p* < 0.01) better than S_1_ for all tuber weight and uniformity traits. Some F_1_ hybrid offspring (15–19%) had better total tuber yield than the best-performing parent. The GEBV accuracy ranged from −0.3928 to 0.4436. Overall, tuber shape uniformity had the highest GEBV accuracy, while tuber weight traits exhibited the lowest accuracy. The F_1_ full sib’s GEBV accuracy was higher, on average, than that of S_1_. Genomic prediction may facilitate eliminating undesired inbred or hybrid offspring for further use in the genetic betterment of potato.

## 1. Introduction

Species having three or more chromosome sets show polyploidy. Polysomic polyploid species arose by the multiplication of chromosome sets from a single or related species [1]. A self-compatible, highly heterozygous, vegetatively propagated tuberous crop, potato (*Solanum tuberosum* L.) is a tetrasomic polyploid (2*n* = 4× = 48 chromosomes) that accumulated during its history of cultivation many deleterious mutations in its genome, which leads to inbreeding depression in the offspring ensuing from self-fertilization; inbreeding depression may also arise from a reduction in intralocus interactions after selfing [2]. Pangenomics revealed that these harmful mutations increased quickly during polyploidization in 4× potato [3]. Crossbreeding has not been able to purge these deleterious mutations from the potato genome. As noted by Zhang et al. [4] who used diploid (2*n* = 2× = 24 chromosomes) potatoes, these mutations, which depend on the cultivar, are often found in the pericentric regions, and most of the deleterious recessive alleles that affect plant survival and vigor are in chromosome regions with high recombination rates. Hence, crossing should be able to purge damaging, large-effect deleterious recessive mutations.

Inbreeding depression reduces fitness because of a likely increase in deleterious homozygous alleles in the offspring after selfing. Inbreeding may lead to fixing favorable alleles to increase overall genetic value, but inbreeding due to drift might increase the frequency of homozygosity for unfavorable alleles and the loss of heterozygosity, thus leading to inbreeding depression [5]. Inbreeding depression affects survival and fertility in offspring derived from related individuals [6]. Furthermore, the effects of inbreeding depression are felt most strongly in the early generations of self-fertilization in polysomic polypoid crops such as alfalfa (*Medicago sativa* L.). It has been argued that this rapid vigor loss results from selfing an alfalfa accession or cultivar with a high frequency of loci bearing three or four distinct alleles [7].

The coefficient of co-ancestry measures the degree of relationship between individuals, while the coefficient of inbreeding tells the probability that two alleles at any locus are identical by descent. Co-ancestry analysis facilitates, therefore, the assessment of relationships among individuals and estimates—through its coefficient—the states of loci in their gametes [8]. Malecot [9] used a probabilistic approach for co-ancestry analysis in diploids, which was further extended by Kempthorne [10] to tetraploids. The inbreeding coefficient in a diploid individual depends on the co-ancestry of its parents, while in a tetraploid, it also includes the inbreeding coefficient of each parent [11]. Furthermore, hybrid offspring derived from partially inbred parents retain some inbreeding in polysomic polyploids. Hence, it is suggested to avoid using inbred parents for breeding quantitative characteristics or using inbred offspring as new cultivars in polysomic polyploid crops due to inbreeding depression.

Inbreeding affects various characteristics in a heterozygous outbreeding crop such as tetrasomic potato, but its effects are complex. Potato tuber yield and seed setting suffer significantly from inbreeding depression [12]. In this regard, Mendoza and Haynes [13] indicated, based on co-ancestry analysis, the genetic similarity of potato cultivars released in the USA between the 1930s and the 1970s, which was further confirmed by the lack of genetic variability for tuber yield using a variance component analysis of multi-site trials over years. A close breeding system also reduces adaptability in potato, as Mendoza and Haynes [13] found in the relatively restricted areas for the US cultivars released in the 1960s and 1970s.

Hybrid vigor or heterosis refers to the greater growth, survival, fertility or yield in the hybrid offspring than in their parents [14]. Mendoza and Haynes [15] postulated an overdominance model for potato tuber yield based on multiple alleles that brings a maximum heterotic value for quadrigenic genotypic structures. They also indicated that to increase the diversity of parents using alien germplasm, sources should first undergo selection for adaptation to photoperiod. However, as demonstrated by Bonierbale et al. [16] with DNA markers, homozygosity was only negatively associated with tuber yield in offspring derived from elite potato breeding germplasm, and maximum heterozygosity was correlated with the yield of large tubers, while maximum heterozygosity did not affect any characteristics in offspring ensuing from crossing elite and alien potato germplasm. Polyploidy offers potato the opportunity to increase both intra- and inter-locus interactions [17], thus maximizing heterosis and explaining why tetraploid potato shows greater tuber yield than diploids [15]. Nonetheless, as noted by Zhang et al. [18], uniform and vigorous F_1_ hybrid diploid offspring may result from crossing inbred pure lines following a genome design. In this approach, the decision making during the inbred line development and crossing them to obtain F_1_ hybrids was based on genome analyses.

Genetic diversity assessments provide insights about the changes in the cultigen pool due to crop improvement. Hirsh et al. [19] demonstrated that crossbreeding scarcely changed the percentage of heterozygosity in potato cultivars released from 1857 to 2011 in North America. It is known that some deleterious mutations may be beneficial under positive selection because of their role in plant diversity and adaptation. There is scant knowledge about the impacts on the heterosis of detrimental mutations [3] that potato acquired during domestication [2], though they may influence many important target traits for its breeding, particularly when it may be possible that strong selection takes advantage of the large mutation effect. It will be worth finding adverse mutations, particularly when they have been ineffectively purged because of the limited recombination in vegetatively propagated crops, while their most recent crossbreeding kept masking them in a heterozygous state [20]. Karunarathna et al. [21] suggest using genomic background selection to select offspring with reduced harmful mutations in a relatively short period in polyploid and asexual crops. Indeed, recombination and further selection reduce genetic load in crops.

Removing deleterious alleles (thus reducing the genetic load) may be a target in vegetatively propagated crops, which often show limited recombination in their genome. For example, Momo and Jannink [22] used a natural selection forward simulator to show that genomic selection—after five generations—could be less effective under a directional dominance model than under an additive model in diploid cassava (*Manihot esculentus*). According to their simulation, although selection increases the frequency of favorable alleles, augmented inbreeding along selection reduces under directional dominance the gain in genotypic values. Purging selection among inbred offspring appears to be only effective in early inbreeding cycles (S_1_ or S_2_) but not in subsequent inbreeding cycles (S_3_ onward) due to the decreasing relatedness of the training set from selection candidates, thus lowering the accuracy (r) of the genomic prediction of breeding values.

The aims of this research were to determine the inbreeding effect in potato tuber yield and other tuber characteristics under a cool, very long daylength by comparing S_0_ (cultivars used as parents), S_1_ (inbreeding of S_0_) and F_1_ (crossing two S_0_) generations and to assess if genomic prediction accuracy is affected by inbreeding in full-sib S_1_ and F_1_ offspring.

## 2. Materials and Methods

The crossing block 2020 of the potato breeding program (Svensk potatisförädling) of the Swedish University of Agricultural Sciences (SLU) included 10 released cultivars and 6 breeding clones that set seeds after crossing by hand. Plants from each cultivar and breeding clone were used for hand-crossing with unrelated pollen or for self-fertilization to obtain F_1_ and S_1_ seed, respectively, at SLU greenhouse in Alnarp (southern Sweden). The cultivars ‘Colleen’, ‘Melody’, ‘Queen Anne’ and ‘Rudolph’ produced S_1_ seed, while there were 28 F_1_ seed, of which 20 were derived from crossing with any of the above four cultivars. Two of these F_1_ were derived from ‘Queen Anne’ × ‘Colleen’ and ‘Queen Anne’ × ‘Melody’.

The field trial at Umeå (63°49′30″ N 20°15′50″ E, Sweden) included the four cultivars (S_0_) producing S_1_ seed, their S_1_ generation and the two F_1_ hybrids noted above among themselves. The field layout was an augmented design [23] with the four S_0_ replicated in 4-plant plots as cultivar checks in the nine incomplete blocks. Each of the nine incomplete blocks also included 100 plots (consisting of four plants) of the S_1_ and F_1_ clones. The spacing was 0.7 m between rows and 0.3 m spacing among plants within the plot. The tubers used for planting ‘Rudolph’ did not sprout well, and this cultivar was not further included for data analysis because of the number of uneven lost plants in each of the nine blocks.

Ten tuber characteristics were evaluated in the field trials, including tuber weight (total and by size: <25, 25–40, 40–50, 50–60 and >60 mm), uniformity of both tuber size and shape, tuber eye depth and tuber flesh reducing sugar, which was measured using potato glucose strips [24]. Tuber uniformity for shape and size as well as tuber eye depth was scored in each plot using a 1–9 scale following Selga et al. [25], which is routinely used by gene banks [26]. Genotype-by-sequencing based on targeting genotyping (https://www.diversityarrays.com/technology-and-resources/targeted-genotyping/, accessed on 19 June 2023) was used for characterizing S_0_, S_1_ and F_1_ with about 2000 SNPs, previously used in genomic prediction of breeding values for cultivars released in western Europe along with Svensk potatisförädling clones [27]. These SNPs, whose chromosome positions are known and spreading throughout the potato genome, were mostly derived from SolCAP SNPs and have a minor allele frequency (MAF) above one in germplasm bred at the Centro Internacional de la Papa (CIP, Lima, Perú) and in the USA. A genome-wide association study revealed that 201 of these SNPs—distributed across the 12 potato chromosomes—had significant marker trait association with tuber weight and other characteristics in the Svensk potatisförädling population (unp. results).

The number of SNPs used for genotyping S_0_, S_1_ and F_1_ sufficed for obtaining GEBVs without losing information [28,29], though rare alleles (with frequency below 1%) were unlikely included in this research. The SNP data had five different allelic stages, which ranged from 0 to 4. In this scale, 0 and 4 are the two homozygotes (OOOO or nulliplex and AAAA or quadriplex), while 1, 2 and 3 refer to simplex (AOOO), duplex (AAOO) or triplex (AAAO) genotypes.

The analysis of variance of the field trial used the following equation:

$$Y_{ij}^{\prime }\;=Y_{ij}-CF$$

where $Y_{ij}^{\prime }$ is the adjusted value of the evaluated characteristic for the ***i***th genotype in the ***j***th block, $Y_{ij}$ is the observed value of the evaluated characteristic, and CF is the correction factor calculated for each block to adjust all phenotypes for each genotype in its respective block. ***CF*** was calculated as

$$CF=\left(\frac{l}{g}\right)\times \left[\underset{i,j}{\sum }Y_{ij}-\underset{i,j}{\sum }\frac{Y_{ij}}{r}\right]$$

where g is the number of check cultivars in each block (i.e., 3), and ***r*** is the number of blocks (i.e., 9). In the analysis of variance of the augmented design, the degrees of freedom for the error should be above 10 following this relationship [30]:

$$r>\left[\frac{10}{c-1}+1\right]$$

where c is the number of cultivar checks. Three contrasts were used for determining inbreeding depression (S_0_ vs. S_1_) and average heterosis (S_0_ vs. F_1_) as defined by Gardner and Eberhart [31] and for comparing inbred and hybrid offspring (S_1_ vs. F_1_).

Genomic predictions and the estimated breeding values (GEBVs) were obtained by fitting the GBLUP model:

y=μ1+u+e

where y is the vector of phenotypes, μ is an intercept, 1 is a vector of ones, u is the random effect of the genotypes, which we assume are distributed as a multivariate normal variable with null mean and variance–covariance matrix $\sigma_{g}^{2}G$, that is, $u∼MN\left(0,\;\sigma_{g}^{2}G\right)$, $\sigma_{g}^{2}$ is the variance parameter associated with the genotypes, and G is a relationship matrix that was computed based on markers using the method proposed by Slater et al. [32] for the full autotetraploid model and implemented in the R package AGHmatrix [33]. Finally, e corresponds to the vector of random errors, $e\sim MN\left(0,\sigma_{e}^{2}I\right)$, with $\sigma_{e}^{2}$ being the variance parameter associated with the errors and u and e being distributed independently.

The prediction ability of the GBLUP model was studied by means of a cross-validation for each trait. Fifty random partitions were generated, 70% of the observations were assigned to the training set and the remaining 30% to the testing set, and Pearson’s correlation coefficient between observed and predicted phenotypes was obtained. Computations were performed using the BGLR package [34] in R [35] using the Bayesian framework. Inferences were based on 15,000 samples obtained after discarding 15,000 samples that were taken as burn-in.

## 3. Results

The 2020 crossing block in Alnarp used 434 of their flowers to obtain 170 berries (ca. 39% crossing success) of 28 unique F_1_ and 4 S_1_ offspring. The berry set was 79%, 82%, 86% and 100% for cultivars ‘Rudolph’, ‘Colleen’, ‘Queen Anne’ and ‘Melody’, respectively. The seed set after the self-fertilization of four cultivars was 113 ± 42, which was lower than the seed set in the F_1_ hybrid offspring (149 ± 29). This result suggested that inbreeding influenced the seed set but not the crossing outcome.

There were significant differences (*p* < 0.05) for all tuber characteristics evaluated (Table 1). The S_0_ parents had greater tuber yield (except for very small sizes) than the inbred offspring (S_1_). ‘Colleen’, ‘Queen Anne’ and ‘Rudolph’ are early-maturity cultivars, while the high-yielding ‘Melody’ is a mid-season cultivar. The average total tuber weight of S_1_ was a quarter less than that of S_0_. This result indicated significant inbreeding depression for this important productivity characteristic of potato. The two F_1_ were on average significantly lower (*p* < 0.001) than the parents, which suggests a lack of average heterosis in the hybrid offspring. Nonetheless, there were some F_1_ offspring (15–19%) whose total tuber yield was in each cross above that of S_0_, thus revealing transgressive segregation for tuber yield in the heterogeneous F_1_. The average total tuber yield of F_1_ was significantly (*p* < 0.001) above that of S_1_. S_0_ had the highest weight among the largest tubers and S_1_ the lowest in a very long day site (about 14.5–ca. 21 h during the cropping season). This result confirmed that inbreeding also affects producing large tubers in potato.

Tuber shape uniformity was not affected by inbreeding, as indicated by the non-significant S_0_ vs. S_1_ contrast (Table 1). The average tuber shape uniformity of the two F_1_ offspring was very similar to their female parent ‘Queen Anne’ and was slightly better than the two male parents (‘Colleen’ and ‘Melody’). However, tuber size uniformity and tuber eye depth were significantly (*p* < 0.001) affected by inbreeding (Table 1), as well as by the parent’s heterozygosity as noted in the segregating F_1_, which on average was significantly (*p* < 0.001) lower for tuber size uniformity and had deeper eyes in the tuber than S_0_. On average, there were non-significant differences (*p* > 0.05) for tuber eye depth between S_1_ and F_1_. The lowest reducing sugars in the tuber flesh were noted in S_0_, which was significantly lower (*p* < 0.01) than in both S_1_ and F_1_ which did not differ (*p* > 0.05) for this characteristic.

Table 2 illustrates the relationships between training and testing sets. The highest genomic prediction accuracy (ρ) estimates (>0.25) for total tuber yield were observed when S_1_ offspring of ‘Colleen’ or ‘Queen Anne’ were the training populations for ‘Queen Anne’ × ‘Colleen’ and ‘Queen Anne’ × ‘Melody’ F_1_s (Table 3). ‘Rudolph’ had the highest ρ estimate (ca. 0.21) for total tuber yield among S_1_s, while ρ estimates were negative in the inbred offspring of ‘Melody’ and ‘Queen Anne’. Most ρ estimates were low (<0.13) for total tuber yield when a non-related population was used for training the GEBV model for either S_1_ or F_1_. The exception was when using Melody S_1_ as training population for predicting total tuber yield in ‘Queen Anne’ × ‘Colleen’. The largest ρ estimates (Table 3) were for tuber shape uniformity (up to 0.4436). S_1_ from ‘Colleen’ and ‘Melody’ were the best for predicting this characteristic in ‘Queen Anne’ × ‘Colleen’ and ‘Queen Anne’ × ‘Melody’ F_1_s, respectively. ‘Rudolph’ S_1_ and ‘Queen Anne’ × ‘Melody’ F_1_ had the highest ρ estimates among S_1_ and F_1_ offspring, respectively. The ρ estimate for non-related offspring was between ‘Rudolph’ S_1_ and ‘Queen Anne’ × ‘Melody’ F_1_. The greatest ρ estimates (>0.20) for tuber size uniformity and tuber eye depth were for ‘Queen Anne’ × ‘Colleen’ F_1_ and ‘Colleen’ S_1_, as well as when using ‘Queen Anne’ S_1_ to predict GEBV for tuber size uniformity in related half-sib ‘Queen Anne’ × ‘Colleen’ F_1_. The best ρ estimates (>0.20) for reducing sugars in the tuber flesh were in ‘Queen Anne’ and ‘Rudolph’ S_1_ as well as for ‘Queen Anne’ and ‘Colleen’ S_1_s predicting the GEBV for this characteristic in their half-sib ‘Queen Anne’ × ‘Colleen’, which had a negative ρ estimate when using F_1_ itself for determining GEBV.

## 4. Discussion

To the best of our knowledge, the novelty of this research includes bringing for the first time a segregating breeding population of tetrasomic polyploid potato to a latitude approaching the Artic Circle (66°14′ N for Norrbotten County in Sweden). Previously, advanced breeding clones, along with released cultivars, were included in field trials at Umeå (Västerbotten County, Norlland, 63°49′30″ N). One of the major concerns for growing potato in such high Nordic latitudes is the adaptation to the short cropping season (about 90 days) under long daylength (14.5–21 h), with relatively low temperature (12.9–16 °C) and mean monthly sunshine hours ranging from 221 to 287. Tuberization, affected by daylength, early maturity and plant growth, as measured by its canopy and vine senescence, is a complex quantitative trait and its multi-genic nature (e.g., circadian clone genes regulating tuberization [36,37] or phytochrome light receptors [38], among others) makes this characteristic difficult to evaluate in the early stages of the potato breeding cycle, particularly in long-day sites with a short cropping season. Furthermore, daylength fluctuations promote flowering time and tuber differentiation plus bulking in potato, both of which, as summarized by Rodríguez-Falcón et al. [39], share common regulatory pathways.

Inbreeding depression significantly affects potato productivity in high Nordic latitudes (Table 1). This result was not surprising because it was also noted in Andean cultivars [40] and in breeding populations of *S. tuberosum* [41] grown under short days in South America. On average, total tuber yield loss due to ID was ¾ of that shown by the released cultivars from which they were derived under the long days of Scandinavia, which was significantly larger than those noted by Golmirzaie et al. [40,41] in Andean cultivars (13%) and in breeding populations of *S. tuberosum* (about 20%) under short days in Peru. It is worth highlighting that ID was only noted in six (out of ten) Andean cultivars [40] and one (out of five) breeding populations of *S. tuberosum* [41], while all cultivars grown under high Nordic latitudes had significant ID. Krantz [42] found that due to ID, a US breeding clone lost ca. 28% of tuber yield, although he indicated that inbreeding ‘produced better parental material than the original parent.’ Furthermore, Krantz and Hutchins [43] advocated inbreeding and selection in the ensuing offspring as a means for the genetic enhancement of potato. However, inbreeding coupled with selection to improve parents has proven to be of limited value for breeding tetrasomic polyploid potato because, as indicated by Mendiburu and Peloquin [44], high-yielding offspring result from maximizing heterozygosity.

The lack of average heterosis for tuber yield in the two F_1_ offspring and the fact that a few breeding clones had tuber yield above that of the highest-yielding parent suggests significant within-family variation for this characteristic. This variation could result from highly heterozygous, diverse parents, which calls for rethinking the concept of specific combining ability (SCA) as a specific individual hybrid combination within the family rather than as the performance of a cross combination versus others. This can be estimated as the deviation of the individual genotypic value from the SCA of the cross.

The visual evaluation of uniformity for tuber shape and size may be influenced by tuber size in potato. The percentage of large tubers decreased due to inbreeding, thus affecting the assessment of tuber shape. Small tubers tend to be round, while large tubers are more distinct and often round and long.

Genomic prediction based on genotyping and together with genome-wide single-nucleotide polymorphisms (SNPs), co-ancestry and phenotypic data is a powerful tool to capture small genetic effects dispersed over the potato genome, thus allowing one to estimate with some accuracy an individual’s breeding value [25]. Allele dosage further improves genomic prediction in polysomic polyploids having a high frequency of distinct heterozygotes and a high dominance degree [28,45], including tetrasomic potato [29]. Nevertheless, Amadeu et al. [46] argued that simpler models based on additive effects are sufficient to obtain GEBVs.

**Figure 1:**
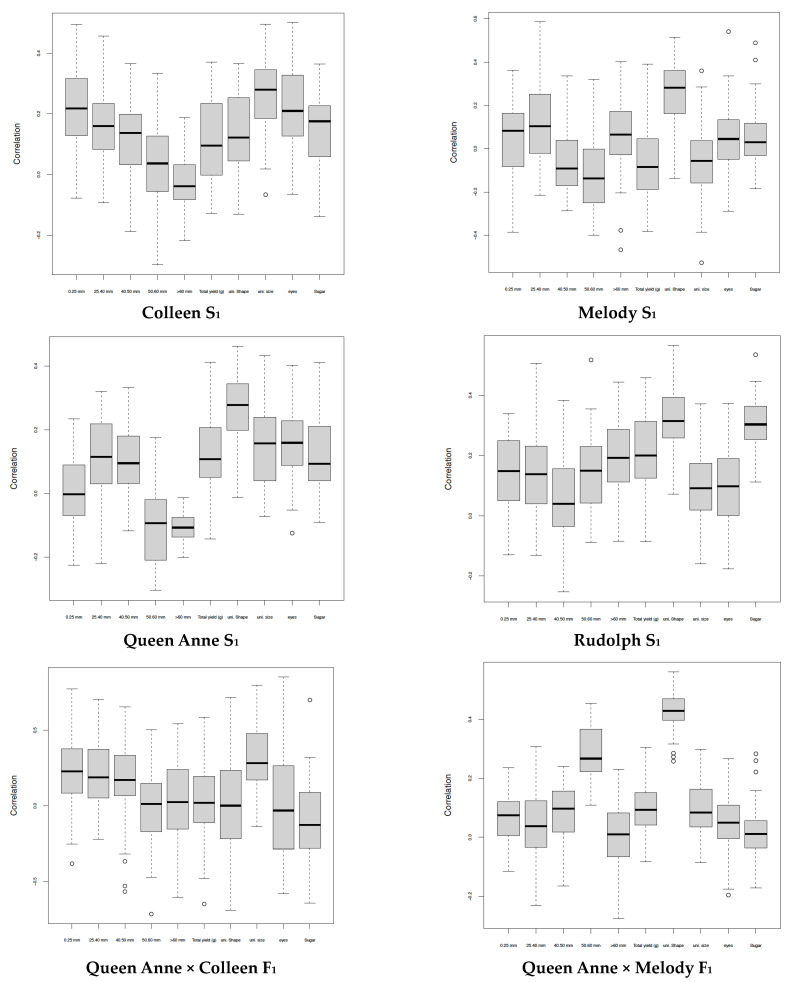
Boxplots showing the accuracy estimates (correlation) of genomic prediction of breeding values for various traits in inbred (S_1_) and hybrid (F_1_) offspring of potato grown at high Nordic latitude (Umeå, 63°49′30″ N 20°15′50″ E, Sweden). From left to right: tuber weight < 25 mm, 25–40 mm, 40–50 mm, 50–60 mm and total; tuber size uniformity; tuber size shape, tuber eyes and reducing sugars in tuber flesh.

Although tuber shape is a monogenic trait in potato, tuber shape uniformity appears to be a low-heritability trait, as estimated by Selga et al. [25] in an advanced breeding population, but it had, on average, the highest genomic prediction accuracy (r) in S_1_ and F_1_ full-sib offspring (Figure 1), as well as when using either half-sib or non-related offspring for across-family validation (Table 3).

On average, reducing sugars in the tuber flesh, which has a medium-high broad-sense heritability [47], had the second largest r estimate in the S_1_ offspring or after across-half-sib-family validation using S_1_ as the training population and F_1_ as the test population. The r estimates for tuber size uniformity in the S_1_ and F_1_ offspring were larger than those of Selga et al. [25] in the advanced breeding population. These r estimates suggest that genomic prediction will be effective for both tuber uniformity traits and for tuber eye depth and reducing sugars in the flesh in S_1_, although r estimates could vary among the S_1_ offspring, perhaps due to the genetic background affected by the deleterious mutation load.

The r estimates for tuber weight were within the range (toward low values) estimated by Endelman et al. [48] and Selga [25] in unselected F_1_ populations. They differed for total tuber yield among the four S_1_ offspring, suggesting that genomic predictions depend on the genetic background and may be effective for purging harmful alleles after inbreeding. Genomic prediction may be further improved in potato when using released cultivars and advanced breeding clones, by using multi-trait, multi-environment GEBV modeling [49]. Modeling genotype × environment interaction in the multi-environment analyses may further exploit the information on the relationship between the site–year combinations, thereby leading to a larger r than those from the single-environment analyses [29], while multi-trait genomic prediction may maximize genetic gain with respect to a focal trait while controlling the variation in multiple secondary traits in potato [49]. Furthermore, deep learning [50] for the genomic prediction of complex multi-genic traits such as tuber yield may be worth pursuing in potato breeding. This approach considers all gene non-additive interactions (dominance and epistasis) that are relevant in polysomic polyploids, thereby improving r, i.e., how reliable a future phenotype of target individuals can be predicted.

Although potato breeders seldom produce S_1_ offspring, Atlin [51] indicated that accurate identification of the value of the alleles they bear is a must to select parents. Selfing along with selection in high-yielding F_1_ offspring might expose deleterious homo-allelic effects and facilitate identifying promising germplasm based on their allelic value. Of course, potential parents derived from this germplasm may become heterozygous after well-thought intercrossing by genome design. Likewise, Atlin [51] stated that the other approach will be to select parents according to their inbred offspring performance. Genomic prediction can be further used to purge deleterious alleles in the breeding population and to select parents for crossing based on their estimated breeding value.

## 5. Conclusions

Inbreeding depression affects tuber characteristics in potato. GEBVs may be useful for eliminating undesired offspring, likely carrying deleterious alleles for productivity, quality and tuber uniformity, in the early stages (e.g., first clonal testing generation or T_1_) of a recurrent selection scheme, but it should be based on related training populations.

## Figures and Tables

**Table 1:** Inbred and hybrid tuber trait performance at a very high Nordic latitude (Umeå, 63°49′30″ N 20°15′50″ E, Sweden).

Cultivar or Offspring	Tuber Weight (4-Plant Plot, g)						Tuber Uniformity ^Z^		Tuber Eye Depth ^Y^	Tuber Flesh Reducing Sugar
	<25 mm	25–40 mm	40–50 mm	50–60 mm	>60 mm	Total	Shape	Size		
Cultivars (S_0_)										
Colleen	34	280	528	1118	810	2771	5.3	5.3	5.0	0.00
Melody	23	452	844	1349	637	3304	5.3	6.6	5.0	0.00
Queen Anne	43	513	1010	721	161	2247	6.1	5.6	6.0	0.22
First inbred generation (S_1_)										
Colleen	39	219	240	176	51	725	5.2	5.2	4.6	0.32
Melody	66	291	298	103	59	781	5.4	4.9	4.9	0.22
Queen Anne	60	249	139	67	11	497	6.7	5.4	5.4	0.47
Rudolph	26	133	248	284	214	889	5.0	4.6	4.0	0.92
Hybrid offspring (F_1_)										
Queen Anne × Colleen	80	257	378	312	136	1162	6.0	4.6	4.7	0.69
Queen Anne × Melody	53	318	497	552	169	1546	6.1	4.7	4.9	0.42
LSD_0.05_	27	104	145	189	136	378	0.9	0.5	0.4	0.41
Statistical significance (*P* > F_c_) of contrasts										
S_0_ vs. S_1_	0.1086	<0.0001	<0.0001	<0.0001	<0.0001	<0.0001	0.8898	<0.0001	<0.0001	0.0031
S_0_ vs. F_1_	0.0008	0.0008	<0.0001	<0.0001	<0.0001	<0.0001	0.0228	<0.0001	0.0002	0.0012
S_1_ vs. F_1_	0.0002	0.0014	<0.0001	<0.0001	0.0059	<0.0001	<0.0001	<0.0001	0.1618	0.3104

^Z^ Scale ranged from 1 (nonuniform) to 9 (uniform). ^Y^ Scale ranged from 1 (deep) to 9 (shallow).

**Table 2:** Training and testing sets for genomic prediction accuracy (r) in inbred (S_1_) and hybrid (F_1_) offspring of potato cultivars.

Training set	Testing sets					
	‘Queen Anne’ S_1_	‘Colleen’ S_1_	‘Melody’ S_1_	‘Queen Anne’ × ‘Colleen’ F_1_	‘Queen Anne’ × ‘Melody’ F_1_	‘Rudolph’ S_1_
‘Queen Anne’ S_1_						
‘Colleen’ S_1_						
‘Melody’ S_1_						
‘Queen Anne’ × ‘Colleen’ F_1_						
‘Queen Anne’ × ‘Melody’ F_1_						
‘Rudolph’ S_1_						

Full sib (S_1_) in yellow; Half-sib (S_1_ and related F_1_) in orange; Unrelated without co-ancestry as per pedigree in light blue.

**Table 3:** Accuracy (ρ) estimates for genomic estimated breeding values for inbred and hybrid full-sib offspring, inbred-hybrid half-sib offspring and non-related offspring.

Training and Validating Offspring	Tuber Weight (4-Plant Plot, g)						Tuber Uniformity		Tuber Eye Depth	Tuber Flesh Reducing Sugar
	<25 mm	25–40 mm	40–50 mm	50–60 mm	>60 mm	Total	Shape	Size		
Full-sibs S_1_ inbred offspring										
A. Colleen	0.227	0.145	0.120	0.032	−0.022	0.105	0.136	0.266	0.217	0.142
B. Melody	0.050	0.119	−0.048	−0.120	0.061	−0.070	0.246	−0.055	0.045	0.056
C. Queen Anne	−0.007	0.143	0.122	−0.196	−0.053	−0.143	0.109	0.186	−0.125	0.218
D. Rudolph	0.137	0.136	0.061	0.138	0.202	0.206	0.316	0.097	0.090	0.297
Full-sibs F_1_ hybrid offspring										
Y. Queen Anne × Colleen	0.218	0.213	0.157	0.002	0.026	0.036	0.009	0.320	−0.001	−0.096
Z. Queen Anne × Melody	0.070	0.049	0.088	0.287	0.011	0.094	0.420	0.095	0.048	0.021
Inbred full-sibs S_1_ (training population)—half-sib F_1_ hybrids (breeding population)										
A.–Y.	0.024	0.083	0.232	0.100	0.111	0.311	0.444	0.104	0.165	0.245
B.–Z.	−0.088	−0.013	0.140	0.059	0.108	0.100	0.217	−0.085	0.060	0.014
C.–Y.	0.221	−0.062	−0.100	−0.152	−0.393	−0.224	0.106	0.310	0.235	0.379
C.–Z.	0.159	0.076	0.156	0.208	0.100	0.264	0.135	0.026	0.030	0.112
Half-sib F_1_ hybrids (one as a training population and the other as a breeding population)										
Y.–Z.	0.100	−0.110	0.141	−0.148	−0.093	−0.057	−0.042	−0.084	0.018	−0.023
Z.–Y.	0.147	−0.153	0.354	−0.266	−0.206	−0.011	0.166	−0.226	0.104	−0.275
Inbred S_1_ (training population)—non-related F_1_ (breeding population)										
A.–Z.	0.096	0.114	−0.078	−0.109	0.056	−0.021	0.066	−0.072	0.076	0.158
B.–Y.	−0.170	0.090	0.079	0.171	−0.151	0.282	0.105	−0.008	−0.082	−0.098
D.–Y.	0.214	0.164	0.173	0.116	−0.037	0.001	−0.040	−0.136	0.130	0.097
D.–Z.	0.106	0.136	0.160	−0.029	0.003	0.123	0.225	0.064	0.024	0.012
Among inbred S_1_ offspring (one as a training population and the other as a breeding population)										
A.–B.	−0.227	−0.074	−0.015	−0.132	0.202	−0.058	0.171	−0.118	0.028	−0.281
A.–C.	0.042	−0.139	−0.096	0.131	0.077	0.093	−0.013	0.035	0.193	−0.077
B.–C.	−0.083	−0.050	0.083	−0.052	0.076	0.099	−0.098	0.166	−0.134	0.185
B.–A.	−0.173	−0.013	0.034	−0.091	−0.014	0.004	0.140	−0.055	0.093	−0.127
C.–A.	0.081	−0.111	−0.125	0.053	−0.002	0.054	0.018	0.116	0.165	−0.083
C.–B.	−0.185	−0.164	0.044	0.108	−0.058	−0.042	−0.076	0.067	−0.028	0.193
D.–A.	0.154	0.148	−0.033	0.048	0.110	0.118	0.230	−0.050	0.094	−0.053
D.–B.	−0.097	−0.151	−0.013	−0.008	0.024	0.014	0.002	−0.066	−0.295	0.146
D.–C.	−0.080	−0.066	0.019	−0.069	0.174	0.030	0.006	−0.179	0.015	0.136

## Data Availability

The datasets used and/or analyzed during the current study are available from the corresponding author on reasonable request.

## Abbreviations

CF: correction factor in augmented design, F_1_: hybrid offspring after crossing two non-sib cultivars, GEBV: genomic estimated breeding value, ID: inbreeding depression, MAF: minor allele frequency, QTL: quantitative trait loci, ρ: genomic prediction accuracy, S_0_: non-inbred cultivar, S_1_: first generation of self-fertilization, SNP: single-nucleotide polymorphism.
